# A Virosomal Malaria Peptide Vaccine Elicits a Long-Lasting Sporozoite-Inhibitory Antibody Response in a Phase 1a Clinical Trial

**DOI:** 10.1371/journal.pone.0001278

**Published:** 2007-12-05

**Authors:** Shinji L. Okitsu, Olivier Silvie, Nicole Westerfeld, Marija Curcic, Andreas R. Kammer, Markus S. Mueller, Robert W. Sauerwein, John A. Robinson, Blaise Genton, Dominique Mazier, Rinaldo Zurbriggen, Gerd Pluschke

**Affiliations:** 1 Molecular Immunology, Swiss Tropical Institute, Basel, Switzerland; 2 INSERM/UPMC UMR S U511, Immunobiologie Cellulaire et Moléculaire des Infections Parasitaires, Faculté de Médecine Pierre et Marie Curie, Centre Hospitalier Universitaire Pitié-Salpêtrière, Paris, France; 3 Pevion Biotech, Bern, Switzerland; 4 Department of Medical Microbiology, University Medical Centre St Radboud, Nijmegen, The Netherlands; 5 Institute of Organic Chemistry, University of Zurich, Zurich, Switzerland; Queensland Institute of Medical Research, Australia

## Abstract

**Objectives:**

Peptides delivered on the surface of influenza virosomes have been shown to induce solid humoral immune responses in experimental animals. High titers of peptide-specific antibodies were also induced in a phase 1a clinical trial in volunteers immunized with virosomal formulations of two peptides derived from the circumsporozoite protein (CSP) and the apical membrane antigen 1 (AMA-1) of *Plasmodium falciparum*. The main objective of this study was to perform a detailed immunological and functional analysis of the CSP-specific antibodies elicited in this phase 1a trial.

**Methodology/Principal Findings:**

46 healthy malaria-naïve adults were immunized with virosomal formulations of two peptide-phosphatidylethanolamine conjugates, one derived from the NANP repeat region of *P. falciparum* CSP (designated UK-39) the other from *P. falciparum* AMA-1 (designated AMA49-C1). The two antigens were delivered in two different concentrations, alone and in combination. One group was immunized with empty virosomes as control. In this report we show a detailed analysis of the antibody response against UK-39. Three vaccinations with a 10 µg dose of UK-39 induced high titers of sporozoite-binding antibodies in all volunteers. This IgG response was affinity maturated and long-lived. Co-administration of UK-39 and AMA49-C1 loaded virosomes did not interfere with the immunogenicity of UK-39. Purified total IgG from UK-39 immunized volunteers inhibited sporozoite migration and invasion of hepatocytes in vitro. Sporozoite inhibition closely correlated with titers measured in immunogenicity assays.

**Conclusions:**

Virosomal delivery of a short, conformationally constrained peptide derived from *P. falciparum* CSP induced a long-lived parasite-inhibitory antibody response in humans. Combination with a second virosomally-formulated peptide derived from *P. falciparum* AMA-1 did not interfere with the immunogenicity of either peptide, demonstrating the potential of influenza virosomes as a versatile, human-compatible antigen delivery platform for the development of multivalent subunit vaccines.

**Trial Registration:**

ClinicalTrials.gov NCT00400101

## Introduction

With over 300 million clinical episodes per year, malaria remains one of the most important infectious diseases in humans [1]. More than 30 years after the first successful protective vaccination of man with attenuated sporozoites, vaccine development against both *Plasmodium falciparum* and *P. vivax* is still ongoing [2], [3]. The most advanced experimental vaccine, RTS,S/AS02A, which is based on the *P. falciparum* circumsporozoite protein (CSP), gave 35% protection against the first episode of malaria and 49% protection against severe malaria for at least 18 month in a clinical trial in Mozambican children [4], [5]. Despite this success it is assumed that a malaria vaccine that is more effective and more cost effective than current malaria control tools, such as insecticide treated bed nets and drug treatment will not be available in the next ten years [6], [7], [8]. It is thought by many that a successful malaria subunit vaccine will have to incorporate antigens against several developmental stages of the parasite. A combination of activities against sporozoites, infected liver cells, merozoites and infected red blood cells may be required to achieve substantial immune protection [9]. Vaccine development against malaria is focusing largely on subunit technologies [9], where the major obstacles include difficulties to retain the native conformation of key antibody epitopes and the need for an effective but safe human-compatible exogenous adjuvant [10]. A main advantage of the subunit approach is that the ideal vaccine will induce immune responses against only those determinants relevant to protection, thus minimizing the possibility of deleterious responses.

We are addressing the problem of protein subunit vaccine design by developing synthetic peptide structures and coupling them to the surface of immunopotentiating reconstituted influenza virosomes (IRIVs) as a liposomal carrier system via a phosphatidylethanolamine (PE) anchor [11], [12], [13], [14], [15], [16]. IRIVs are spherical, unilamellar vesicles, prepared by detergent removal from a mixture of natural and synthetic phospholipids and influenza surface glycoproteins. Hemagglutinin, a membrane glycoprotein of the influenza virus mediates binding to sialic acid on target cells and is a fusion-inducing component, facilitating antigen delivery to immunocompetent cells. IRIVs represent a universal antigen-delivery system for multivalent subunit vaccines, since antigens can be either attached to their surface to elicit antibody and CD4 T cell responses or encapsulated in their lumen to elicit CD8 T cell responses [13], [17]. They have an excellent safety profile and two virosomal vaccines (against influenza and hepatitis A virus) are already registered for human use in more than 40 countries [18].

We are optimizing synthetic peptides in an iterative selection process to develop vaccine components with native-like conformation that elicit high titers of parasite cross-reactive antibodies [11], [12], [13], [14], [15], [16], [19], [20]. Peptides are synthesized from antigens that (i) have a documented and essential role in parasite development, (ii) have secondary structure motifs suggesting surface exposition, (iii) have conserved sequence stretches, and (iv) induce parasite-inhibitory antibodies. Based on these criteria we try to choose protein domains containing protection-relevant epitopes, thus avoiding the induction of deleterious immune responses as observed during infection with *P. falciparum*. Lead peptides are optimized in a step-wise process. The key readout are the parasite-binding properties of antibodies elicited in mice after immunization with the corresponding peptide coupled to the surface of IRIVs. After preclinical profiling in experimental animals two candidate peptides, i.e. AMA49-C1, a 49-aa cyclic peptide derived from loop I of domain III of *P. falciparum* apical membrane antigen 1 (AMA-1) [13], and UK-39, a conformationally constrained cyclic peptide containing five NPNA repeats derived from the central repeat region of *P. falciparum* CSP [15], have been tested in a phase 1a clinical trial. Virosomal formulations of AMA49-C1 (designated PEV301) and UK-39 (designated PEV302) were both safe and elicited anti-peptide IgG in all volunteers immunized with an appropriate peptide concentration [21]. In this report we focus on the detailed immunogenicity data for this first clinical trial with PEV302. Complete results for PEV301 will be presented elsewhere. While mean ELISA titer development for the different vaccination groups has been described previously [21], we analyze and correlate here anti-peptide ELISA titers with titers of parasite cross-reactive antibodies and with parasite-inhibitory activities at the level of individual sera. Moreover, we show affinity maturation of UK-39-specific antibodies induced by vaccination.

## Materials and Methods

The protocol for this trial and supporting CONSORT checklist are available as supporting information; see Checklist S1 and Protocol S1.

### Participants

Serum samples were collected during a phase 1a clinical trial at the Clinical Research Center, University Hospital, Basel, Switzerland. Details of the study design are described in a separate report [21]. Briefly, a prospective phase 1a, single blind, randomized, placebo controlled, dose-escalating study was conducted in 46 healthy malaria-naïve adult volunteers.

Eligible study participants were randomized into six groups receiving virosomal formulations of the following antigens: 10 µg AMA49-C1 (group A, n = 8), 10 µg UK-39 (group B, n = 8), 50 µg AMA49-C1 (group C, n = 8), 50 µg UK-39 (group D, n = 8), 50 µg AMA49-C1+50 µg UK-39 (mixture of separate virosome-preparations containing one antigen each) (group E, n = 8) or empty IRIVs serving as controls (group F, n = 6). In this report we only used samples from PEV302 immunized individuals and controls.

### Vaccine

Two virosomally-formulated vaccines, PEV301 (incorporating the AMA-1 derived PE-peptide conjugate AMA49-C1 [13]) and PEV302 (incorporating the CSP derived PE-peptide conjugate UK-39 [15]) were produced according to the rules of GMP. Tests for sterility, pyrogenicity, immunogenicity in animals, stability and chemical composition were performed on the vaccine lots used in this trial. Each dose was composed of 10 or 50 µg AMA49-C1 or UK-39, 10 µg *Influenza* hemagglutinin, 100 µg phospholipids and PBS ad 0.500ml. Volunteers immunized with a combination of virosomally formulated AMA49-C1 and UK-39 received a double dose of influenza proteins. The vaccine was stored at 4°C. The immunization regimen was 0, 2 and 6 months. The trial vaccines were administered *i.m.* in the left arm on day 0, in the right one on day 56–65, and in the left again on day 175–186.

### Immunogenicity

Blood sampling for antibody titer measurement by ELISA, IFA and Western blot was done at screening visit (baseline and 1^st^ vaccination), at the days of the 2^nd^ and 3^rd^ vaccination, 21 days after each vaccination and one year after the third vaccination (groups B and E only). Serum from volunteers with no history of malaria was used as a negative control and serum from an individual living in a malaria endemic area was used as a positive control for IFA and Western blotting studies.

### ELISA

Standard ELISAs were performed as described before [13]. Briefly, Polysorp™ microtiter plates (Nunc, Dr. Grogg, Stetten-Deiswill, Switzerland) were coated with 10 µg/ml AMA49-C1 or UK-39 in PBS. After blocking, plates were incubated with two-fold serial dilutions of human serum. Horseradish-peroxidase-conjugated goat anti-human IgG antibodies (KPL, Socochim, Lausanne, Switzerland) were used as secondary antibodies and 1,2-Diaminobezene substrate (OPD) (Fluka, Sigma, Buchs, Switzerland) in citrat-buffer (4mg/ml OPD)+0.01% H_2_O_2_ for development. The endpoint titer is the last serum dilution where the OD_test sera_≥2×OD_negativ serum pool_. The negative serum pool is from 10 non-immunized malaria-naïve individuals living in Switzerland.

### NH_4_SCN elution ELISA

Avidity ELISA analyses with peptide-PE conjugates were performed essentially as described before [22]. After coating and blocking, as described for standard ELISA, serum samples were added in triplicates at constant dilutions (approx. halfmax titer). After a wash step, plates were incubated 15min with NH_4_SCN diluted in 0.1M NaH_2_PO_4_ buffer (pH 6) at the following molarities: 5M, 4M, 3M, 2M, 1M, 0.5M, 0.25M. Control wells were incubated with 0.1M NaH_2_PO_4_ buffer without NH_4_SCN. After washing, plates were incubated with alkaline phosphatase-conjugated affinity-pure F(ab́)_2_ fragment goat anti-human IgG (Fc-specific) antibodies (Jackson Immuno Research Laboratories, West Grove, PA) and developed with phosphatase substrate solution. The avidity index corresponds to the NH_4_SCN concentration (M) eluting 50% of the bound antibodies.

### Indirect immunofluorescence assay (IFA)

IFAs were performed as described before [15]. Air-dried unfixed *P. falciparum* (strain NF54) salivary gland sporozoites attached to microscope glass slides were incubated with serial dilutions of sera and detected with Cy™3-conjugated affinity-pure F(ab́)_2_ fragment goat anti-human IgG (Fc-specific) antibodies (Jackson Immuno Research Laboratories, West Grove, PA) diluted in blocking solution containing 0.01 mg/ml Hoechst dye no. 33256 (Sigma, St. Louis, MO).

### SDS-PAGE and immunoblotting

*Anopheles stephensi* salivary gland lysate containing about 100,000 *P. falciparum* sporozoites was separated on a 10% SDS PAGE mini-gel and transferred to a nitrocellulose filter as described before [13]. Filter strips were incubated with serial dilutions of sera. Monoclonal antibody EP9 served as a positive control [14]. Antibodies were detected with alkaline peroxidase-conjugated affinity-pure F(ab')_2_ fragment goat anti-human IgG (Fc-specific) antibodies (Jackson Immuno Research Laboratories, West Grove, PA) Blots were developed using ECL™ Western blotting detection reagents (Amersham Biosciences, Buckinghamshire, England).

### *P. falciparum* in vitro invasion inhibition assay

Blinded inhibition assays were performed as described before [23]. Briefly, triplicate primary human hepatocyte cultures were inoculated with *P. falciparum* sporozoites (1×10^5^/Lab-Tek well). After 3h at 37°C, cultures were washed, further incubated in fresh medium for 3 days, and fixed in methanol. Quantification of exoerythrocytic forms was done by immunofluorescence. To determine the effects of anti-UK-39 antibodies on sporozoite infectivity, sporozoites were incubated with hepatocytes in the presence of affinity purified polyclonal human IgG from immunized volunteers. Inhibition was determined in comparison to PBS control.

### *P. falciparum* migration inhibition assay

Blinded migration inhibition assays were performed as described before [24]. Briefly, HepG2 cells were incubated with sporozoites in the presence of cell-impermeant tracer fluorescein isothiocyanate (FITC)-dextran. After 2 hours at 37°C cells were washed to remove extracellular FITC-dextran and analyzed by flow cytometry. Inhibition was determined in comparison to PBS control.

### Statistical analysis

Statistical significance of increases in avidity was tested using a Wilcoxon signed-rank test to compare avidity indices between different time points. Correlation of titers in immunogenicity assays and correlations of titers with inhibition was analyzed with nonparametric (Spearman) correlation. Pearson correlation coefficients were used to assess correlation of invasion and migration inhibition. Average titers are displayed as geometric means. The level of significance used was 0.05 for all analyses. No adjustments were made for multiple comparisons. All statistical analyses and graphs were made using GraphPad Prism version 4.03 for Windows, GraphPad Software, San Diego, CA.

## Results

Details on study design, safety and basic immunogenicity data of this trial are reported in a separate study [21].

### Outcomes and estimation

#### Development of IgG responses against the synthetic antigen UK-39

**Figure 1:**
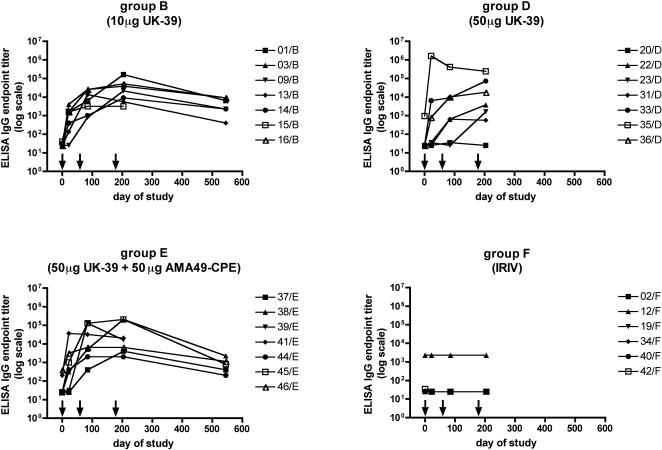
ELISA endpoint titers. Shown are anti-UK-39 IgG ELISA endpoint titers for individual volunteers of all vaccination groups at baseline (0) and 21 days after each vaccination and one year after the third vaccination. Arrows indicate days of immunization. Only the groups immunized with 10 µg UK-39 or 50 µg UK-39+50 µg AMA49-C1 were followed up for one year.

Already after two immunizations, all volunteers receiving the virosomal formulation of 10 µg UK-39 alone (group B) or 50 µg UK-39 in combination with 50 µg of AMA49-C1 (group E) had developed an anti-UK-39 IgG response with an endpoint titer above 400 in ELISA (Figure 1), as shown before [21]. In group D receiving 50 µg of UK-39 alone, five out of seven volunteers (71%; exact binomial 95% CI = 29%, 96%) had sero-converted after two immunizations with titers above 600. A further increase in IgG titer after the third immunization was observed only in some volunteers in all vaccination groups. A one-year follow-up was performed with one group for each of the two antigens and for the combination. One year after the third immunization titers had dropped, but all volunteers of groups B and E remained positive (endpoint titer >10^2^) in ELISA. No increase in anti-UK-39 antibody titers after vaccination was observed in volunteers of the control group F receiving empty IRIVs (Figure 1). The overall highest endpoint titer (>10^6^) was observed after the first immunization in a volunteer (35/D) from the 50 µg UK-39 group, which had a pre-immunization ELISA endpoint titer of about 10^3^ (Figure 1).

**Figure 2:**
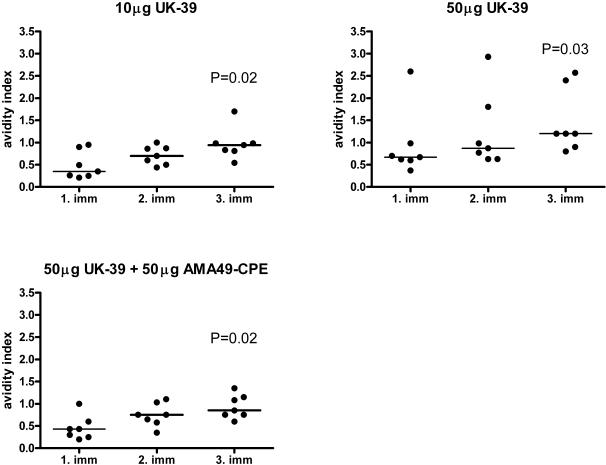
Affinity maturation of vaccine-induced IgG. Median of avidity indices for the anti-UK-39 IgG responses in all vaccination groups receiving UK-39 three weeks after the first, second and third immunization. The avidity index is the NH_4_SCN concentration (M) where 50% of the bound antibodies are eluted. Shown are results obtained with individual sera and the median (line) for each time point. Wilcoxon signed-rank test was used to calculate the statistical significance of a difference in avidity between the third and the first vaccination.

Antibody affinity maturation is a key event during memory B cell development. We measured the avidity of anti-UK-39 IgG in elution ELISAs with chaotropic salt. The mean avidity of the vaccine-induced IgG responses gradually increased by two-fold in all groups over the course of immunization (Figure 2). Volunteer 35/D who had pre-existing UK-39 cross-reactive antibodies had high-affinity anti-UK-39 IgG already after the first immunization.

### Ancillary analyses

#### Sporozoite cross-reactivity of anti-UK-39 IgG responses

**Figure 3:**
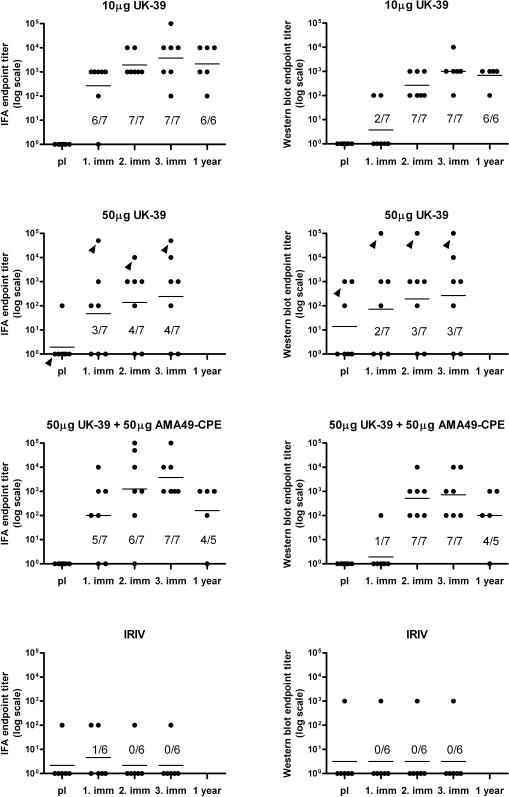
Cross-reactivity of vaccine-induced IgG with *P. falciparum* sporozoites. IFA (left panel) and Western blotting (right panel) were performed with *P. falciparum* salivary gland sporozoites. Shown are endpoint titers and number of volunteers showing an increase in titer compared to pre-immune (pI) serum. Note that the volunteers in the 50 µg group who gave positive results before the first immunization showed no increase in titer upon vaccination. Blood samples were taken pre-immune, three weeks after the first, second and third immunization and one year after the third immunization. Lines represent the geometric mean of all volunteers in the respective group. Arrows indicate volunteer 35/D that had a pre-existing anti-UK39 IgG ELISA titer of 10^3^. Only the groups immunized with 10 µg UK-39 or 50 µg UK-39+50 µg AMA49-C1 were followed up for one year.

After three immunizations all volunteers (14/14) immunized with 10 µg UK-39 or 50 µg UK-39 combined with 50 µg AMA49-C1 and five of seven volunteers (71%; exact binomial 95% CI = 29%, 96%) immunized with 50 µg UK-39 alone showed a vaccine-induced increase in IgG that was cross-reactive (endpoint titers ≥10^2^) with *P. falciparum* sporozoites in IFA and in Western blotting (Figure 3). Mean IFA and Western blotting endpoint titers increased with each of the three immunizations. One year after the third immunization ten out of eleven (91%; exact binomial 95% CI = 59%, 99.8%) volunteers in the 10 µg UK-39 and the combination group were still positive (endpoint titers ≥10^2^). IRIVs alone (group F) did not elicit parasite cross-reactive IgG; volunteer 12/F who had exhibited a persistent anti-UK-39 ELISA titer already present before the first vaccination also had an IFA and Western blotting titer.

Statistical analysis of ELISA, IFA and Western blotting titers (from all volunteers and time-points measured) revealed a close correlation between all assays. Spearman's rank correlation coefficients were r = 0.892 (P<0.0001) for the comparison of titers in ELISA and IFA, r = 0.703 (P<0.0001) for the comparison of titers in ELISA and Western blot, and r = 0.649 (P = 0.0001) for the comparison of titers in IFA and Western blot.

**Figure 4:**
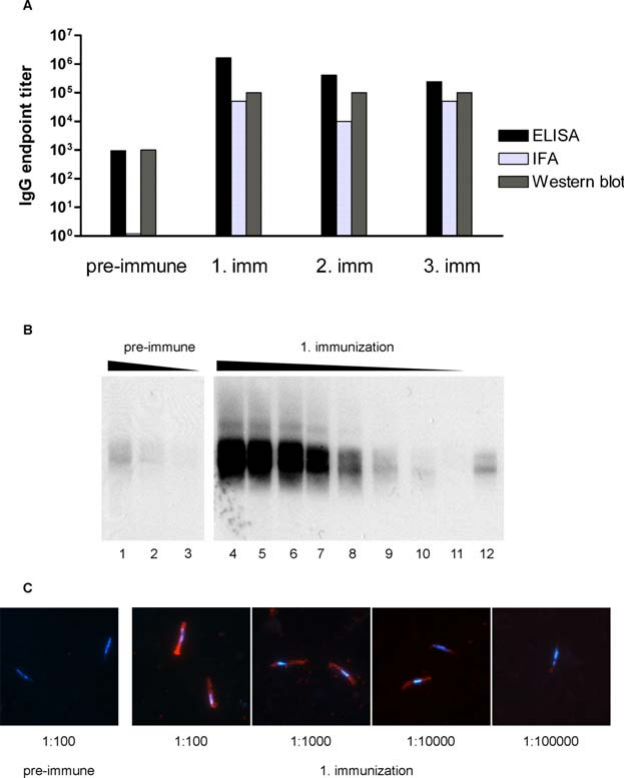
Boost of a pre-existing anti-UK-39 cross-reactive antibody response in volunteer 35/D. A: Comparison of ELISA, IFA and Western blotting titers of pre-immune serum and sera taken three weeks after each immunization. Shown are representative titers from one out of three experiments. B: Western blotting results obtained with serum taken before and three weeks after the first immunization. Dilutions are: 1, 1∶200; 2, 1∶400; 3, 1∶800; 4, 1∶3,000; 5, 1∶9,000; 6, 1∶27,000; 7, 1∶81,000; 8, 1∶243,000; 9, 1∶729,000; 10, 1∶2,187,000; 11, 1∶6,561,000; 12, Positive control anti-NANP mAb EP9. C: IFA on *P. falciparum* sporozoites with serum taken before and three weeks after the first immunization. An overlay of DNA staining with Hoechst dye no. 33256 (blue) and Cy3-immunofluorescence staining (red) is shown.

Volunteer 35/D, who had a pre-immune ELISA titer that strongly increased by just one immunization, also developed very high IFA and Western blotting titers (10^5^) after the first immunization (Figure 4A). While this volunteer had a pre-existing Western blotting (Figure 4B) and ELISA titer of 10^3^, no IFA staining was observed with the pre-immune serum (Figure 4C).

### Sporozoite inhibitory activity

Anti-sporozoite antibodies can interfere with critical steps of sporozoite development like migration through tissues [24], [25] and invasion of hepatocytes [26]. To test the impact of anti-UK-39 antibodies on sporozoite infectivity we used two different in vitro assays measuring sporozoite migration through hepatocytes (HepG2 cell line) and sporozoite invasion of hepatocytes in the presence of purified IgG from immunized volunteers.

**Figure 5:**
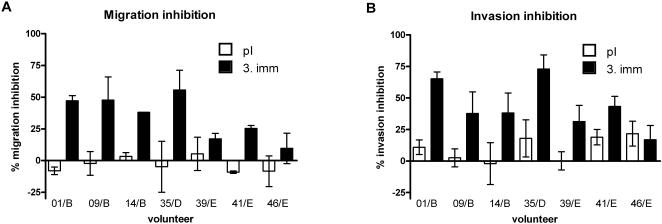
Functional activity of anti-UK39 IgG responses. Sporozoite migration and invasion inhibition by IgG preparations (final concentration 1 mg/ml) purified from sera taken before the first (pI) and three weeks after the third immunization (3.imm). Three volunteers (xy/B) immunized with 10 µg UK-39, one volunteer (35/D) immunized with 50 µg UK-39, and three volunteers (xy/E) immunized with 50 µg UK-39+50 µg AMA49-C1 were chosen. Shown are the means and standard deviation from at least two independent experiments. A: In vitro inhibition of sporozoite migration through human HepG2 cells. B: In vitro Inhibition of sporozoite invasion of primary human hepatocytes.

Migration inhibition experiments were performed as described by Mota et al. [24]. Sporozoites were incubated with HepG2 cells in the presence of cell-impermeant FITC-dextran. Wounded cells take up FITC-dextran, which is trapped in the cell cytoplasm after wound resealing and can be detected by flow cytometry. Total IgG from nine volunteers from all immunization groups representing the whole range of observed immune responses (including controls) were purified. The presence of purified total IgG (final concentration 1mg/ml) from UK-39-immunized volunteers reduced sporozoite migration in a dose dependent manner. Vaccination-induced increases in migration inhibitory activity were observed in all seven UK-39-immunized volunteers (Figure 5A). There was a trend to higher rates of inhibition in group B compared to group E immunized with the combination vaccine. This observation was statistically significant (two-sided unpaired t test, P = 0.0005) but not relevant due to the small sample size. IgG preparations from control individuals, immunized with AMA49-C1 or IRIVs alone showed no vaccine-induced inhibition of sporozoite migration (data not shown).

The impact of antibodies on sporozoite invasion can be determined in invasion inhibition assays where the development of liver stage parasites after incubation of primary human hepatocyte cultures with *P. falciparum* sporozoites is quantified. Vaccine-induced inhibition of sporozoite invasion was seen with purified IgG (final concentration 1mg/ml) from six out of the same seven volunteers immunized with UK-39, and analyzed for migration inhibition (Figure 5B). No effect of dose and formulation of the vaccine on the inhibitory potential of the induced antibodies was observed. IgG preparations from controls immunized with AMA49-C1 or IRIVs did not inhibit sporozoite invasion (data not shown). Boosting of a pre-existing UK-39 cross-reactive antibody response in volunteer 35/D (Figure 4) induced IgG with high migration and invasion inhibitory potential showing no difference to IgG preparations of other UK-39-immunized volunteers (Figure 5A and B).

**Figure 6:**
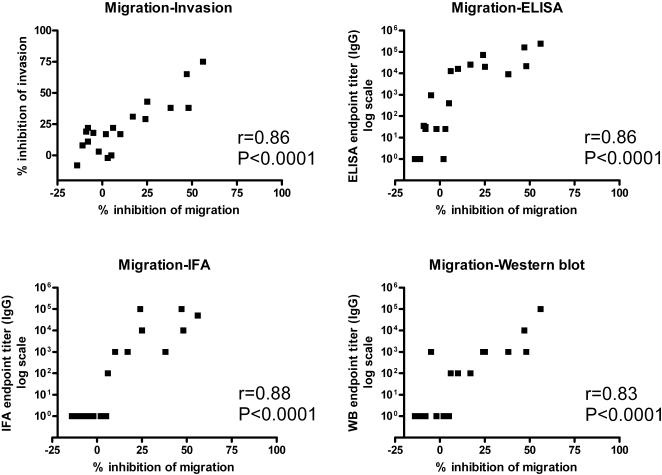
Correlation (r) of functional activity with antibody titers. Sera taken before the first and three weeks after the third immunization from eight PEV302 immunized volunteers plus one IRIV control and one control from group C immunized with PEV301 alone (only post immunization) analyzed in inhibition assays were included in the analysis. Pearson correlation coefficients were calculated for correlation of invasion and migration inhibition; nonparametric (Spearman) correlation was used for analysis of immunogenicity assays.

We found a strong correlation (Pearson correlation r = 0.8648, P<0.0001) between inhibition of invasion and inhibition of migration by IgG preparations (Figure 6). To determine a relation between this functional activity and the immunogenicity results we also compared IgG titers measured in ELISA, IFA and Western blotting with inhibition results. IgG titers measured in all three immunogenicity assays closely correlated with the migration and invasion inhibitory activity of the antibodies (data not shown for invasion inhibition) (Figure 6).

## Discussion

### Interpretation

In this report we describe immunological and functional properties of antibodies elicited by the virosomally formulated synthetic peptide UK-39 in a phase 1a clinical trial. Since virosomally formulated UK-39 molecules represent repeat structures on the surface of IRIVs, they have the potential to amplify the antigen-specific B cell pool enormously, allowing a very early and efficient switch to a long-lived IgG response [27], [28]. Two immunizations with 10 µg UK-39 were indeed enough to induce high titers of peptide-specific IgG in all volunteers [21]. A higher dose of 50 µg peptide delivered with the same amount of IRIVs was not superior in terms of sero-conversion and mean antibody titers elicited. However, when co-administered with a second virosomal vaccine component, the 50 µg dose of UK-39 was as immunogenic as the 10 µg dose alone. This may be related to the doubling of the amount of influenza antigens administered with the combination vaccine and the adjuvant-effect associated with the viral components. All volunteers had pre-existing influenza T cell and antibody responses, but no correlation with the magnitude of the responses against the malaria antigens were observed (Peduzzi et al., personal communication).

One volunteer (35/D) of the 50 µg UK-39 group needs special attention. Pre-immune serum of this volunteer already contained antibodies that were reactive with UK-39 in ELISA and with CSP in Western blotting. This cross-reactivity, which was not related to a known history of malaria exposure, was exceptionally boosted already by the first immunization, yielding the highest titers observed in the entire trial. Even though the observed pre-existing humoral response was not malaria-related, this suggests that IRIV-based vaccines might have the potential to boost natural immunity. It is therefore highly likely, that two or even one vaccine dose may be sufficient to boost pre-existing immunity in vaccine recipients from endemic areas.

We found a close correlation between IgG titers against the peptide UK-39 in ELISA and IgG titers in IFA and Western blotting with whole parasites and parasite-derived antigens, respectively. Moreover titers in these immunogenicity assays were closely correlating with in vitro inhibition of sporozoite migration and invasion of hepatocytes. This confirms the observation that the conformationally restrained peptide UK-39 is very close to the natural conformation of the NANP repeats, as already indicated by NMR analysis of UK-39 and the crystal structure of an NPNA repeat [15], [29].

Vaccine induced immunity has to include formation of immunological memory, the key to successful immune protection [30]. One crucial step of memory formation is antibody affinity maturation in germinal centers where high-affinity variants of antigen-specific B cells are selected for entry into the long-lived memory B cell compartment [31]. We found an increase in anti-UK-39 antibody avidity over the course of immunization in all three vaccine groups indicating memory B cell formation. The mean avidity index was highest in group D receiving 50 µg of UK-39 due to particularly high avidity indices in two individuals (35/D and 36/D). One year after the third immunization all volunteers receiving the 10 µg dose of UK-39 had remained positive in ELISA, Western blotting analysis and IFA supporting the hypothesis that vaccination with virosomally formulated UK-39 has induced long-term IgG production by long-lived plasma cells.

One of the major problems associated with malaria vaccine development are missing in vitro and in vivo correlates of protection. Various in vitro assays and experimental animal models are available to test functional activity of induced responses, but the predictive value of these systems cannot be determined until the results are correlated with clinical efficacy of vaccine candidates [32]. No in-depth study comparing in vitro results, animal studies, artificial human challenge and vaccine efficacy in endemic areas has been reported to date. Furthermore, standardized assays to evaluate functional activity of vaccine-induced antibodies with a validated set of control sera would be most desirable for future malaria vaccine development.

Induction of antibodies inhibiting in vitro sporozoite migration, growth, and development was observed in a majority of UK-39-immunized volunteers tested, regardless of vaccine formulation. Inhibition was IgG concentration-dependent and confirmed data obtained with animal sera [15]. Inhibition of sporozoite migration and invasion was observed at concentrations of total purified IgG from immunized volunteers that were more than ten times lower than the physiological IgG concentration of about 13 mg/ml. Even though this inhibition might only be partial and would not prevent the development of blood-stage infection, previous experience with sporozoite vaccines and insecticide treated bednets has shown that reduction of merozoites released from the liver can decrease the occurrence of severe forms of disease [5], [33]. It will be crucial to follow-up these inhibition results in phase 2a challenge trials and efficacy studies in field trials to validate these in vitro inhibition assays as correlates for protection.

Invasion inhibition was positively correlated with migration inhibition, suggesting that anti-UK-39 antibodies interfered with parasite gliding motility, which is based on the same mechanism of redistribution of proteins along the sporozoite surface as host cell invasion [34]. Whether inhibition is due to a specific block of a component of the machinery necessary for gliding or by steric hindrance is difficult to elucidate. Based on the observed close correlation of results in invasion inhibition experiments and the far easier migration inhibition assays, the migration inhibition system might be a valuable second line method to measure the biological activity of anti-CSP antibodies. Further advantages of the migration inhibition assays are the reduced time needed, the option for quantitative analysis by flow cytometry and the possibility to perform the experiments with hepatocyte cell lines. Altogether, these properties make the migration inhibition assay an interesting tool for screening large numbers of serum samples in clinical trials.

### Overall evidence

These results demonstrate the suitability of IRIVs as a human-compatible carrier/adjuvant system for the induction of potent humoral immune responses against synthetic peptide antigens. The CSP-specific humoral immune responses characterized in this report were very uniform, cross-reactive with *P. falciparum*, affinity maturated, long-lived and parasite-inhibitory. Combination of two components had no negative effect on the elicited immune response against the single components, showing the potential of influenza virosomes as a versatile antigen delivery platform for multivalent vaccine development.

## Supporting Information

Checklist S1 CONSORT Checklist
(0.05 MB DOC)

Protocol S1 Trial Protocol
(0.32 MB PDF)
